# Metformin as a Potential *In Vitro* Anticancer Modulator of Adenosine Monophosphate Kinase: A Review

**DOI:** 10.1155/2024/1094274

**Published:** 2024-08-27

**Authors:** A. G. R. Greshamali Jinadasa, H. M. Kasuni Akalanka, N. D. Amal Wageesha, Sagarika Ekanayake

**Affiliations:** ^1^ Department of Basic Sciences Faculty of Allied Health Sciences University of Sri Jayewardenepura, Gangodawila, Nugegoda, Sri Lanka; ^2^ Rural Health Research Institute Charles Sturt University Orange, Orange, NSW 2800, Australia; ^3^ Department of Biochemistry Faculty of Medicine Sabaragamuwa University of Sri Lanka, PO Box 01, Hidellana, Ratnapura, Sri Lanka; ^4^ Department of Biochemistry Faculty of Medical Science University of Sri Jayewardenepura, Gangodawila, Nugegoda, Sri Lanka

## Abstract

Metformin (MET) is the commonly prescribed hypoglycemic agent used in the treatment of type 2 diabetes mellitus (DM). Pleiotropic effects of MET are emerging as a medication for other diseases including breast cancer (BC). Therefore, a literature review was conducted to investigate whether the anticancer effects of MET are mediated through adenosine monophosphate kinase (AMPK). This review assessed published data focusing on studies where BC cell lines were treated with MET to explore its potential anticancer effects via AMPK on BC cells. The published data reveals that activated AMPK induces anticancer effects primarily by suppressing cell proliferation, induction of apoptosis, and cell cycle arrest, inhibition of metastasis and invasion, alteration of tumor microenvironment, and downregulation of tumorigenesis. In addition, MET was observed to induce AMPK-mediated effects when combined with other drugs. Further studies on assessing the potential use of MET alone or in combination with other drugs would pave the way to design new treatment strategies for BC.

## 1. Introduction

Metformin (MET) is identified as the most effective molecule for glycemic control [1]. It is a synthetic biguanide which is commonly prescribed as a medication to reduce elevated plasma glucose levels. Biguanides are biomolecules derived from *Galega officinalis* which is a herbaceous plant in the subfamily Faboideae of the legume family Fabaceae [2]. However, due to the induced lactic acidosis by natural members of the biguanide family, MET was introduced as a synthetic glycemic control with reduced acidity at physiological pH compared to its natural members [3, 4]. Metformin is a known hydrophilic molecule, and its active form in pharmaceutical drugs is metformin hydrochloride. Upon oral administration, MET is absorbed via plasma membrane monoamine transporters (PMAT) and organic cation Transporters 2 and 3 (OCT 2 and 3) in the intestine. Following absorption OCT 1 and multidrug and toxin extrusion 1 (MATE1) of the liver supports its hepatic uptake [3]. Primary site of action of MET is the liver where it reduces hepatic gluconeogenesis. In addition, MET reduces glucose absorption in the intestine, increases insulin sensitivity, and reduces plasma glucose concentration [5]. MET has been observed to increase the peripheral cellular uptake of glucose and inhibit Complex I of the mitochondrial electron transport chain, resulting in decreased ATP levels thus affecting the cellular energy homeostasis [6]. According to the National Library of Medicine USA, no evidence is available on the potential metabolism of MET nor its potential binding to specific plasma proteins. The molecule is excreted in its original form by renal elimination [7, 8].

In recent years, scientists have identified additional therapeutic benefits of MET, particularly pharmacological significance for other diseases including polycystic ovarian syndrome (PCOS), cardiovascular diseases, renal diseases, liver disease, cancers including breast, prostate, colorectal, pancreatic, endometrial, and hematological melanoma [4, 9–11]. These data indicate the potential use of MET as an anticancer drug together with currently available cancer treatment strategies in addition to its use in glycemic control. According to the National Cancer Institute, cancer is the uncontrolled proliferation of cells, which arises due to one or many genetic mutations. Mutations are common in highly proliferative tissues. Check points of the cell cycle, correct these mutations in healthy cells and the abnormal cells are destroyed by the immune system. However, in cancer, mutations occur in proto-oncogenes coding for proteins that function at these cell cycle checkpoints, tumor suppressor genes, or the mutations activate protooncogenes. In cancer, one or more of the corrective mechanisms are defective, and the cells are subjected to uncontrolled growth. If not treated, cancer cells could invade the nearby tissue or even undergo metastasis. Breast tissues are highly subjected to cell proliferation with the exposure to cyclic changes in the reproductive cycle in women. This makes the breast tissue more vulnerable for mutations. Among the various cancers that exhibit therapeutic responses to MET, breast cancer (BC) stands out as notably significant with its therapeutic response. It is the most common carcinoma and is the leading cause of death due to carcinoma among women worldwide. Global cancer statistics reported 2.3 million new BC cases in 2020, which accounts for 11.7% of total carcinoma incidence [10]. According to epidemiological studies, one in eight women has a risk of developing BC during their lifetime. Thus, the identification of effective treatment strategies for BC is of immense importance. BC occurs due to a variety of modifiable and nonmodifiable risk factors. Diabetes mellitus (DM) is a well-studied risk factor for its concurrent behavior with BC [10]. Studies have proven that DM enhances the relative risk of BC incidence by 10%–20% [10]. Therefore, the concurrent prognostic behavior of MET on BC reveals its potential therapeutic use.

Cancer cells are rapidly dividing cell masses with high demand for nutrients and energy. For the survival of cancer cells, glucose is of paramount importance as the primary source of energy. Thus, these cells are found to be dependent on glycolysis to fulfil their energy requirements for the growth and synthesis of metabolites required for cell proliferation [12]. Therefore, hyperglycemia is a favorable environmental factor for carcinogenesis. This implies that lowering glucose concentration would have an impact on lowering the risk of cancer incidence. Several *in vitro*, preclinical, and clinical studies reported the anticancer efficacy of MET individually or in combination with chemotherapeutic drugs in the treatment of different forms of BC [13, 14]. The BC patients prediagnosed with DM, who received MET during their neoadjuvant chemotherapy, have shown a higher pathologically complete response rate than DM patients who did not receive MET [11]. These shreds of evidence prove the potential impact of MET as an anticancer agent.

As stated previously, MET inhibits the mitochondrial complex I in the electron transport chain and alters energy homeostasis by decreasing ATP:AMP ratio. Decreased ATP:AMP ratio activates adenosine monophosphate kinase (AMPK) [6]. The molecule AMPK is known as the master metabolic kinase in cells [15]. Given that cancer cells elicit increased demand for energy for their rapid growth and survival, altered energy homeostasis by MET suggests its potential AMPK-mediated anticancer effects. Therefore, an electronic database search was conducted on PubMed using key terms “Metformin, breast cancer, cell line, AMPK, adenosine monophosphate kinase.” The search focused on freely accessible data evaluating the potential anticancer effects of MET, mediated by AMPK, on BC cell lines. Upon activation, AMPK has been shown to inhibit uncontrolled cell proliferation, induce apoptosis, suppress metastasis and invasion, reduce tumorigenesis, and alter the tissue microenvironment, manifesting various anticancer effects. The molecular mechanisms underlying these pathways are discussed in detail in the current review.

## 2. Metformin and Activated AMPK

**Figure 1:**
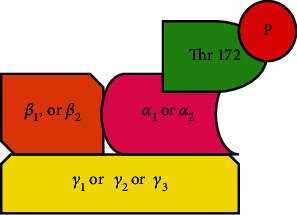
Activated adenosine monophosphate kinase (AMPK) trimeric molecule with *α*, *ß*, and *γ* subunits.

Metformin indirectly activates AMPK due to the alteration of ATP:AMP ratio [16]. A MET concentration of at least 1 mM is said to be required to trigger AMPK activation in cultured cells [17]. AMPK is a trimeric molecule with *α*, *β*, and *γ* subunits (Figure 1). Alfa is the catalytic subunit, while *β* and *γ* are regulatory subunits [18]. There are seven gene products that code for these three subunits with two *α* (*α*_1_ and *α*_2_), two *β* (*β*_1_ and *β*_2_), and three *γ* (*γ*_1_, *γ*_2_, and *γ*_3_) molecules, respectively. As such, 12 different trimeric AMPK molecules are formed by different combinations of the above seven molecules.

The phosphorylation of threonine residue (Thr172), in *α* subunit and canonical regulation by adenine nucleotides including AMP and ADP, activates the molecule [19]. Reduced glucose levels decrease cellular ATP resulting in decreased ATP:AMP ratio. Further, AMP allosterically modulates AMPK followed by phosphorylation of Thr172 of *α* subunit. The activated AMPK, also termed as phosphorylated AMPK (p-AMPK) acts by up or downregulating different cellular regulatory processes. The exact mechanisms of action of AMPK are not yet fully understood.

When considering the BC cell lines, the cellular uptake of MET is dependent on the number of cationic transporters in the cell membrane [6]. MET-mediated activation of AMPK is observed in drug-resistant and sensitive BC cell lines [20]. In addition, the glucose concentration of the cell culture medium also alters the *in vitro* activation of AMPK via MET mediated pathway [21, 22]. Activated p-AMPK upregulates glucose uptake via glucose transporter type 4 (GLUT-4), glycolysis, fatty acid oxidation, autophagy, and mitochondrial biogenesis. In addition, it downregulates cell growth and proliferation, protein synthesis, cell cycle progression [19], fatty acid synthesis, glycogen synthesis [4], and nucleotide synthesis [23]. In an overview, p-AMPK shifts the cellular metabolism towards reduced energy consumption and increased energy generation [19]. As such in a carcinogenic environment, p-AMPK induces the availability of cellular energy which is favorable for metabolically active cells. Also, in contrast, p-AMPK inhibits cell cycle progression and cellular anabolic reactions which retard the growth and proliferation of cancer cells. These effects collectively contribute to inhibiting cancer progression, as evidenced by clinical studies. Thus, it is timely to evaluate the data on *in vitro* anticancer behavior of p-AMPK, which could expose similar or new cellular mechanisms that establish AMPK as a potential anticancer modulator.

## 3. Antiproliferative and Apoptotic Effects

Cancer cells are mutated cells with rapid cell proliferation. Inhibition of cell proliferation is an effective target in designing therapeutics for cancers. In BC cell line studies, AMPK-induced antiproliferative effects have been identified. Evidence in the literature states that activated AMPK exerts inhibition of metadherin (MTDH), dishevelled segment polarity protein 3 (DVL3), Wnt/*β*-catenin pathway, and also the mammalian target of rapamycin (mTOR) [6, 22, 24–34] proving its antiproliferative effects.

MTDH is an oncogenic protein, expressed in cancer cells that induce cellular proliferation, metastasis, and chemoresistance. The inhibition of MTDH was observed in triple-negative BC (TNBC) cells. The activation of MTDH is induced by myelocytomatosis (c-Myc) which is a master oncogene code for proteins that regulate proliferation and apoptosis. Overexpression of c-Myc increases cellular proliferation [28]. The action of c–Myc is inhibited by glycogen synthase kinase 3*β* (GSK3*β*), a serine protein kinase. It phosphorylates and inhibits the enzyme glycogen synthase thus interrupting the conversion of UDP-glucose to glycogen. Interestingly AMPK is an activator of GSK3*β*. This supports that AMPK downregulates anabolic mechanisms in a cell and shifts the cellular metabolism away from energy utilization towards energy-yielding processes. Therefore, activated AMPK downregulates MTDH via inhibition of c-Myc in GSK3*β*-mediated pathway [24].

Another significant antiproliferative observation is the AMPK-mediated antifolate chemotherapeutic activity induced by MET. When BC cell lines were treated with MET, 5-formimino-tetrahydrofolate accumulated. This is a tetrahydrofolate which carries one carbon metabolite essential for *de novo* purine and pyrimidine synthesis. Accumulation of this folate implies interruption of one carbon supply to the *de novo* nucleotide synthesis, proving the antifolate activity of MET. Even though direct involvement of MET is observed in this antifolate activity, AMPK-mediated mechanism is observed secondarily via other molecules. Ataxia–telangiectasia mutated protein kinase (ATM) is a regulatory protein of DNA damage response. Activation of ATM retards the progression of carcinoma towards the invasive phase. In addition, ATM is a known upstream kinase of AMPK and induces tumor suppressor pathways. Following the antifolate action of MET, ATM-mediated AMPK activation has been observed as a secondary pathway. Therefore, in BC cells, MET-mediated activation of ATM/AMPK tumor suppressor in a secondary pathway induces antiproliferative properties [27].

A well-known antiproliferative mechanism is the arrest of cell cycle progression. MET is also observed to induce cell cycle arrest via the AMPK-mediated pathway [28]. Activated AMPK affects cell proliferation through cell cycle arrest by reducing the levels of the DVL3 and Wnt/*β*-catenin pathways. DVL3 is expressed in BC cells and is upregulated by oncogene c-Myc and cyclin D. Overexpression of DVL3 upregulates *β*-catenin and, thus, induces Wnt/*β*-catenin pathway which induces *in vitro* cell proliferation in BC cells. However, MET-mediated activation of AMPK downregulates DVL3 and *β*-catenin, thereby arresting the cell cycle [21]. In addition, GSK3*β*-mediated inhibition of c-Myc also amalgamates the antiproliferative mechanisms *in vitro* [24]. Thus, the overall effect is MET-induced AMPK-mediated antiproliferation via cell cycle arrest in BC cells *in vitro* [21].

### 3.1. Effects on mTOR Pathway

The mechanistic target of rapamycin or the mammalian target of rapamycin (mTOR) is the master controller of protein synthesis in cells. Activated AMPK inhibits the mTOR pathways. The active form of mTOR upregulates protein synthesis and is involved in multiple signaling pathways and regulates cell proliferation, apoptosis, and autophagy. Inhibitors of mTOR use a unique mechanism in blocking the translation of proteins, thus enhancing their abilities as effective anticancer agents. Simultaneously, these inhibitors block tumorigenesis [29]. Hence, *in vitro* mTOR-mediated anticancer effects have been observed in drug-sensitive and drug-resistant BC cell lines [22, 24–26]. The mTOR has two complexes mTORC1 and mTORC2, where mTORC1 is associated with the initiation of protein translation, while mTORC2 activates some growth factor receptors and regulates endocytosis. Activated AMPK is a negative regulator of mTOR which is mediated via the tuberous sclerosis complex 1/2 (TSC1/2) pathway [30]. Consequently, inhibition of the mTOR by AMPK is an important anticancer regulatory pathway [6, 31]. In addition, treatment of 5–30 mM of MET on preheated (42°C) MCF-7 and MDA-MB-231 BC cells was observed to phosphorylate AMPK and reduce the phosphorylation of mTOR, thus reducing the activation of mTOR [32]. Another profound AMPK-mediated inhibition of mTOR was observed when BC cells were treated with everolimus together with MET [30]. A similar combined effect on inhibition of the mTOR pathway has been observed with ursolic acid and MET [33]. These results might be due to the synergetic action of MET with these molecules. Even though these results are supporting the mTOR inhibition by MET-activated AMPK, contrasting findings suggest that mTOR inhibition is mediated via activated protein kinase Akt and not AMPK [22, 31]. Akt is a group of serine/threonine kinases which mediate major cellular mechanisms including cell cycle progression, genome stability, glucose metabolism, and protein synthesis [34]. Thus, it is questionable whether the inhibition of mTOR is AMPK-dependent. Further, experiments are suggestive in the identification of the mTOR/AMPK axis and its contribution to mediating anticancer effects.

## 4. Downregulation of Tumorigenesis

Tumorigenesis is the process of gradual loss of normal cellular properties while increasing the malignant properties. Inhibition of this process is a salient target in anticancer treatment. Transforming growth factor-*ß* (TGF-*ß*) is considered a tumor suppressor in the early stage of tumorigenesis. In contrast, in the later stage of cancer, it promotes metastasis. Therefore, elevated serum TGF-*ß* levels are an indication of metastatic cancer in BC patients [35]. Phosphorylated AMPK is observed to suppress the transcription of TGF-*ß*. Interestingly, DNA analysis reveals suppression of TGF-*ß* promoters in MET-treated cells [35]. Therefore, *in vitro* combined effect of AMPK and MET might negatively regulate metastasis controlled by TGF-*ß* facilitating better prognosis.

## 5. AMPK-Mediated Epithelial–Mesenchymal Transformation

Activated AMPK suppresses the malignant transformation of the tissue microenvironment. Transformation of cellular structures, including cell receptors, chromatin content, and augmentation of nuclear:cytoplasmic ratio, is the malignant transformation of cancer cells. Healthy cells undergo numerous changes, and these changes facilitate their survival and migration. Epithelial–mesenchymal transition (EMT) transformation of BC cells is one such mechanism which ensures its survival and distant metastasis. EMT of BC cells is TGF-1 mediated. Upon EMT cell invasion of the basement tissues, metastasis to distant locations is initiated. When BC cells were treated with MET, EMT was suppressed by activated AMPK [36]. Another proposed mechanism of AMPK action is reversing EMT by maintaining mesenchymal phenotypes. Allosteric AMPK activator, OSU-53 mediates the suppression of phenotypic mesenchymal transformation. This suppression is mediated through the activation of the Foxo3*α* regulator. Foxo3s are master regulators which induce gene expression in response to environmental stimuli. OSU-53 activates Foxo3*α*, whereby it mediates the suppression of EMT through two Akt-dependent pathways. The first, through cytoplasmic sequestration of murine double minute 2 (MDM2), inactivates Foxo3*α*. The second, through Akt-mediated, reduced phosphorylation of Foxo3*α*, leading to its nuclear translocation. These two mechanisms together mediated the AMPK-dependent Foxo3*α*-activated repression of EMT [15].

## 6. AMPK-Mediated Changes in Tumor Microenvironment

Intercellular, as well as cell and tissue environment communication, is equally important for the growth and development of healthy and carcinogenic tissues. Alterations of the tumor microenvironment are known to induce carcinogenesis and tumor metastasis. In addition, MET induces the regulation of the tumor-associated macrophage (TAM) population towards the antitumor phenotype. The TAM possesses controversial effects on the tumor microenvironment through different phenotypes. They are of two subtypes TAM termed as Types 1 and 2. Type 1 is also identified as M1 and is present in nonmalignant or regressing tumors. These cells express surface antigen CD16. They are involved in the promotion of tumor lysis through proinflammatory activity. The other subtype is M2 (Type 2), where M2 expresses cell surface receptor CD206. Type 2 is present in malignant tumors and enhances tumor growth by producing cytokines and negatively regulating antitumor immune responses. When the TAM population was treated with MET, gene expression and cytokine analysis proved the induced skew of the antitumor phenotype. MET-treated BC cell lines have induced the secretion of M1-inducing cytokines such as IL-12 and TNF-*α* and the controversial effect observed on M2-related cytokines IL-8, IL-10, and TGF-*β*. In addition, MET-treated BC cells have inhibited their cytokines secretions IL-4, IL-10, and IL-13 which promote M2 phenotype and conversely upregulate the M1 phenotype-inducing cytokines.

MET-activated AMPK directly phosphorylates and modulates nuclear factor-*κ*B (NF-*κ*B) activity through the phospo-p65 subunit. Through AMPK-NF-*κ*B signaling pathway, a population shift towards M1-type macrophages in a cytokine-mediated environment is observed [37]. This evidence suggests MET-induced potential immune reactions to induce antitumor effects *in vitro*, which might be further evidence for potential anticancer modulation of AMPK.

The bidirectional communication between cancer-associated fibroblasts and the tumor cells has been observed to aid in cancer progression and invasion in BC. This communication is altered and inhibited by MET-induced p-AMPK, thus retarding tumor invasion. Cellular secretions or expressions of molecules facilitate cellular communication. Cancer cells secrete hypoxia-inducible factor (HIF) which is a marker responsible for vascularizing the ischemic tissue environment. Vascularization improves the survival of ischemic tissue. Upon exposure to the BC cell environment, cancer-associated fibroblasts increase the expression of hypoxia-inducible factor-1*α* (HIF-1*α*). The expression HIF-1*α* by cancer-associated fibroblasts is inhibited and degraded by increased levels of p-AMPK activated by MET through the transformation of tumor fibroblasts in the prolyl hydroxylases axis [38]. These alterations of the MET mediated by p-AMPK further emphasize the role of AMPK as an anticancer modulator *in vitro.*

Another noteworthy finding is the effect of glucose concentration on the culture media. The glucose concentration of the media is observed to have a direct correlation with the survival of BC cells. It is proven through stimulation of AMPK-mediated anticancer actions of MET on normoglycemic and hypoglycemic states studied. Data further confirms that high glucose levels provide a favorable environment in BC progression which is clinically evident with the concurrent behavior of DM and BC [39]. Thus, these *in vitro* data provide evidence of underlying causes for the increased BC incidence rates among uncontrolled DM patients and also provide a clue for the better prognosis of patients on MET-controlled plasma sugar levels.

## 7. Potential Combined Effects With Other Drugs

Synergism is the interaction of two or more drugs when their combined effect is greater than the sum of the effects seen when each drug is given alone. Interestingly, MET is observed to depict profound activation of AMPK when treated in combination with anticancer and some conventional drugs. Low concentrations of tamoxifen (5 *μ*M) and MET (5 mM) when introduced to BC cells have significantly inhibited DNA synthesis, cell proliferation, cell growth, and induced apoptosis [40]. In suggestive of another synergetic action, combined treatment of paclitaxel and MET induced higher AMPK phosphorylation in tumors with TNBC [13, 14]. This induced activation of AMPK would facilitate profound elevation of AMPK-mediated anticancer effects on treated cells discussed above. In addition, MET, when treated with aspirin, has induced apoptosis both in TNBC and endocrine-sensitive BC models *in vitro* predicting synergistic effects [41, 42]. Interestingly, MET with vitamin D also has induced anticancer mechanisms via AMPK-mediated pathways [43]. Consequently, it is suggestive that MET is acting as a potential synergistic partner for selected anticancer drugs. In addition, uncover the anticancer properties of other biomolecules through AMPK mediation. Thereof, AMPK is a potential anticancer modulator.

## 8. Other

**Figure 2:**
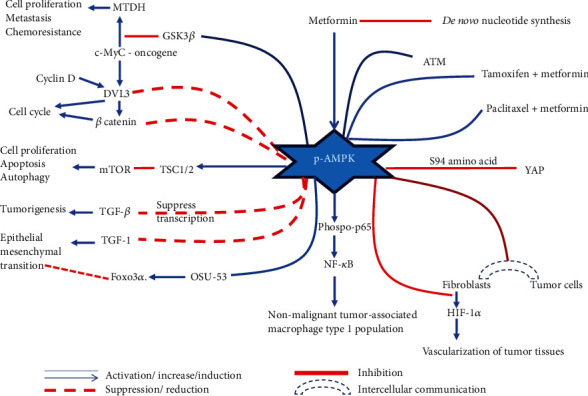
Anticancer effects of activated adenosine monophosphate kinase (AMPK: adenosine monophosphate kinase; ATM-ataxia–telangiectasia-mutated protein kinase; c-Myc: myelocytomatosis; DVL: dishevelled segment polarity protein 3; EMT: epithelial–mesenchymal transformation; HIF-1*α*: hypoxia-inducible factor-1*α*; MET: metformin; MTDH: metadherin: mTOR: mammalian target of rapamycin: NF-*κ*B: nuclear factor-*κ*B: TGF-*ß*: transforming growth factor-*ß*; TSC: tuberous sclerosis complex; YAP: yes-associated proteins).

Beyond what is discussed above, yes-associated protein (YAP), which is a key transcriptional regulator/coactivator in controlling growth, tissue homeostasis, and carcinogenesis, is also identified as another anticancer pathway. Nuclear localization of cytoplasmic YAP regulates gene expression and induces cell proliferation. Activation of this regulatory pathway is common in drug-resistant cancer cells. AMPK directly phosphorylates YAP through S94 amino acid residue and inhibits its activity on cell growth and carcinogenesis. This MET-induced YAP inhibition is observed in drug-sensitive and resistant BC cells *in vitro* [20]. However, not all the anticancer mechanisms of MET are mediated by AMPK [44]. The molecular mechanisms and the changes in the cellular environment are tabulated (Table 1) In summary, this review focuses on the MET-induced AMPK-mediated in vitro anticancer mechanisms on BC cell lines (Figure 2).

## 9. Conclusion

MET-mediated anticancer mechanisms alter BC cell survival mainly by affecting cellular processes, including antiproliferation, apoptosis and inhibition of mTOR pathway. Furthermore, MET influences downregulation of tumorigenesis, AMPK-mediated epithelial–mesenchymal transformation, changing the tissue microenvironment, and by potential combined effects with other drugs. Further studies on the impact of MET on BC will be beneficial when developing therapeutic targets.

## Figures and Tables

**Table 1:** AMPK-mediated anticancer mechanisms.

**Section**	**Mechanism**	**References**
Antiproliferative and apoptotic effects	Metadherin (MTDH) inhibition in triple-negative BC (TNBC) cells.	Miller et al. [27]
	Activation of DNA damage regulatory response via ataxia–telangiectasia-mutated protein kinase (ATM)/AMPK pathway	Miller et al. [27]
	Cell cycle arrest via dishevelled segment polarity protein 3 (DVL3) and Wnt/*β* catenin pathways	Zou et al. [21]
	Glycogen synthase kinase 3*β* (GSK3*β*)-mediated inhibition of myelocytomatosis (c-Myc)	Gollavilli et al. [24]

Effects on mTOR pathway	Inhibitors of mTOR block tumorigenesis.	Zhuang et al. [22] Gollavilli et al. [24] Queiroz et al. [25] Cha et al. [26]
	Negative regulation of mTOR path via tuberous sclerosis complex 1/2 (TSC1/2) pathway by AMPK	Wang et al. [30]
	Phosphorylation of mTOR reduced by activated AMPK in preheated MCF7 and MDA-MB-231 cells	Lee et al. [32]
	Inhibition of mTOR when treated with everolimus together with MET	Wang et al. [30]
	Inhibition of mTOR when treated with ursolic acid and MET	Zheng G et al. [33]

Downregulation of tumorigenesis	Suppression of transcription of transforming growth factor-*β* (TGF-*β*) by MET in AMPK pathway	Drabsch and Dijke [35]

AMPK-mediated epithelial–mesenchymal transformation	Tumor growth factor 1-mediated epithelial–mesenchymal transformation induced by MET via AMPK pathway.	Li et al. [36]
	OSU-53 mediates the suppression of phenotypic mesenchymal transformation via cytoplasmic sequestration of murine double minute 2 (MDM2) and Akt-dependent Foxo3*α* inactivation and phosphorylation through the AMPK pathway.	Chou et al. [15]

AMPK-mediated changes in tumor microenvironment	Alteration of tumor-associated macrophage population and modulation of nuclear factor-*κ*B (NF-*κ*B) pathway through AMPK-mediated mechanisms in the tumor microenvironment.	Chiang et al. [37]
	Inhibition of the expression of hypoxia-inducible factor 1 in cancer-associated fibroblasts by AMPK	Shao et al. [38]
	Glucose concentration of the environment facilitates cancer cell growth and survival. This data is evident with concurrent behavior of diabetes mellitus with BC	Zordoky et al. [39]

Potential combined effects with other drugs	Tamoxifen (5 *μ*M) and MET (5 mM) synergistically inhibit DNA synthesis, cell proliferation, cell growth, and induced apoptosis *in vitro*.	Ma et al. [40]
	Paclitaxel and MET synergistically phosphorylate AMPK in TNBC cells *in vitro*. Thus, it induces AMPK-mediated anticancer cellular processes.	Rocha et al. [13] Cai, Everett, and Thakker [14]
	Aspirin and MET synergistically induce anticancer effects on ER-positive and TNBC cells *in vitro*	Amaral et al. [41] Zhao et al. [42]
	MET and vitamin D induce AMPK-mediated anticancer effects	Guo et al. [43]

Other	AMPK-mediated phosphorylation of yes-associated protein (YAP) through S94 amino acid residue to inhibit cell growth and proliferation in carcinogenesis.	Liu et al. [20]

## Data Availability

Data related to the manuscript is available upon request from the corresponding author.
